# Isolated Langerhans cell histiocytosis in the stomach of adults: four-case series and literature review

**DOI:** 10.1007/s12308-024-00584-9

**Published:** 2024-04-23

**Authors:** Jianmin Zhao, Yanlei Li, Yanlin Zhang, Xue Mei, Wei Liu, Yinghong Li

**Affiliations:** 1 Department of Pathology, Beijing Friendship Hospital, Capital Medical University, No. 95, Yong’an Road, Xicheng District, Beijing, China; 2 Department of Pathology, Tianjin Medical University, Tianjin, China

**Keywords:** Adult, BRAF V600E, Gastric, Gastrointestinal, Langerhans cell histiocytosis

## Abstract

Langerhans cell histiocytosis (LCH) of the stomach is rare. Moreover, it is usually found in pediatric patients with systemic diseases and may be associated with a poor prognosis. Solitary gastric LCH in adults is extremely rare and is often misdiagnosed or missed. The aim of our study was to review cases of gastric LCH and explore the characteristics of the disease further. A retrospective study of all patients admitted with solitary gastric LCH was conducted between 2013 and 2023. Clinical manifestations, endoscopic and pathological features, immunophenotypes, and molecular changes were collected from medical records. We examined four cases (one female, three males) of gastric LCH. The affected patients were between 33 and 70 years of age. Endoscopically, three patients presented with a solitary polyp or elevated lesions, whereas one patient showed no abnormalities. Under a microscope, all cases showed abnormal proliferation of histiocytoid cells infiltrating in a nested or sheet-like fashion. The tumor cells were medium-sized, with a slightly eosinophilic cytoplasm, irregular or renal-shaped nuclei, folded nuclear membranes, visible nuclear grooves, and the infiltration of inflammatory cells in the background. Immunohistochemically, all lesions expressed CD1a, S-100, langerin, and cyclinD1. One case showed diffuse BRAF V600E positivity. Follow-up data were available for all patients from 4 to 36 months, and all patients were alive without recurrence or progress at the time of manuscript preparation. Combined with previously reported data, solitary adult gastric LCH is more common in male patients, most of whom are asymptomatic or exhibit only mild gastrointestinal symptoms, with a good prognosis. Endoscopy often reveals solitary polyps or protruding lesions; rare cases may progress to multifocal/multisystem lesions, necessitating long-term close follow-up.

## Introduction

Langerhans cell histiocytosis (LCH) is a rare histiocytic disease characterized by abnormal proliferation of bone marrow-derived Langerhans cells. Its presentation is unpredictable and ranges from solitary lesions of a single system to multiorgan disease with severe organ dysfunction [1]. LCH usually affects children and rarely adults. The most frequent body parts involved in adult LCH are the bone, lung, skin, and the pituitary/hypothalamus [2, 3]. LCH involving the stomach in adults is extremely rare and has been described in only a limited number of cases [4–18].

To enhance our understanding of the disease among clinicians and pathologists, we examined four cases of adult solitary gastric LCH and reviewed the relevant literature to explore its clinical manifestations, endoscopic and pathological features, immunophenotypes, molecular changes, and differential diagnosis.

## Materials and methods

### Materials

This study was approved by the Institutional Review Board. Four cases of solitary adult gastric LCH, with detailed follow-up information, were identified in the surgical pathology files (encompassing the years 2013–2023), including three cases from Beijing Friendship Hospital and one case from Tianjin Medical University General Hospital. Clinical manifestations, endoscopic features, pathological features, immunophenotypes, and molecular changes were reviewed. Clinical follow-up data were obtained for all patients, over a period from 4 to 36 months after the initial diagnosis.

### Immunohistochemistry (IHC)

IHC staining was performed on deparaffinized 3 μm-thick slices with the EnVision method. The antibodies used in our study included those against langerin (12D6, ready-to-use, Zhongshan Gold Bridge), CD1a (010, 1:100 Maxim), S-100 (4C4.9, 1:200, Maxim), pan-cytokeratin (AE1/AE3, 1:200, Maxim), Ki67 (MXR002, 1:200, Maxim), cyclinD1 (SP4, 1:100, Maxim), CD68 (KP1, 1:300, Maxim), HMB45 (1:100, Maxim), and BRAF V600E (VE1, ready-to-use, Ventana). Appropriate positive and negative controls were used simultaneously. Two experienced pathologists assessed the IHC staining.

### Molecular detection

Fluorescence polymerase chain reaction (PCR) was used to identify the mutation status of *BRAF*, *NRAS*, and *KRAS*, according to the protocol of the human *BRAF/NRAS/KRAS* mutation detection kit (Xiamen Amoy Biopharmaceutical Co., Ltd., Xiamen, China). The *BRAF* gene mutations detected by the kit included exon 15 (*V600E1*, *V600E2*, *V600D1*, *V600D2*, *V600K*, and *V600R*). The *KRAS* gene mutations detected by the kit included exon 2 (*G12S*, *G12D*, *G12C*, *G12R*, *G12V*, *G12A*, *G13C*, and *G13D*), exon 3 (*Q61L*, *Q61R*, and *Q61H)*, and exon 4 (*K117N*, *A146T*, *A146V*, and *A146P*). The *NRAS* gene mutations detected by the kit included exon 2 (*G12D*, *G12S*, *G13R*, *G12C*, *G12V*, *G12A*, and *G13V*), exon 3 (*Q61R*, *Q61K*, *Q61L*, and *Q61H*), and exon 4 (*A146T*).

## Results

### Clinical findings

**Fig. 1:**
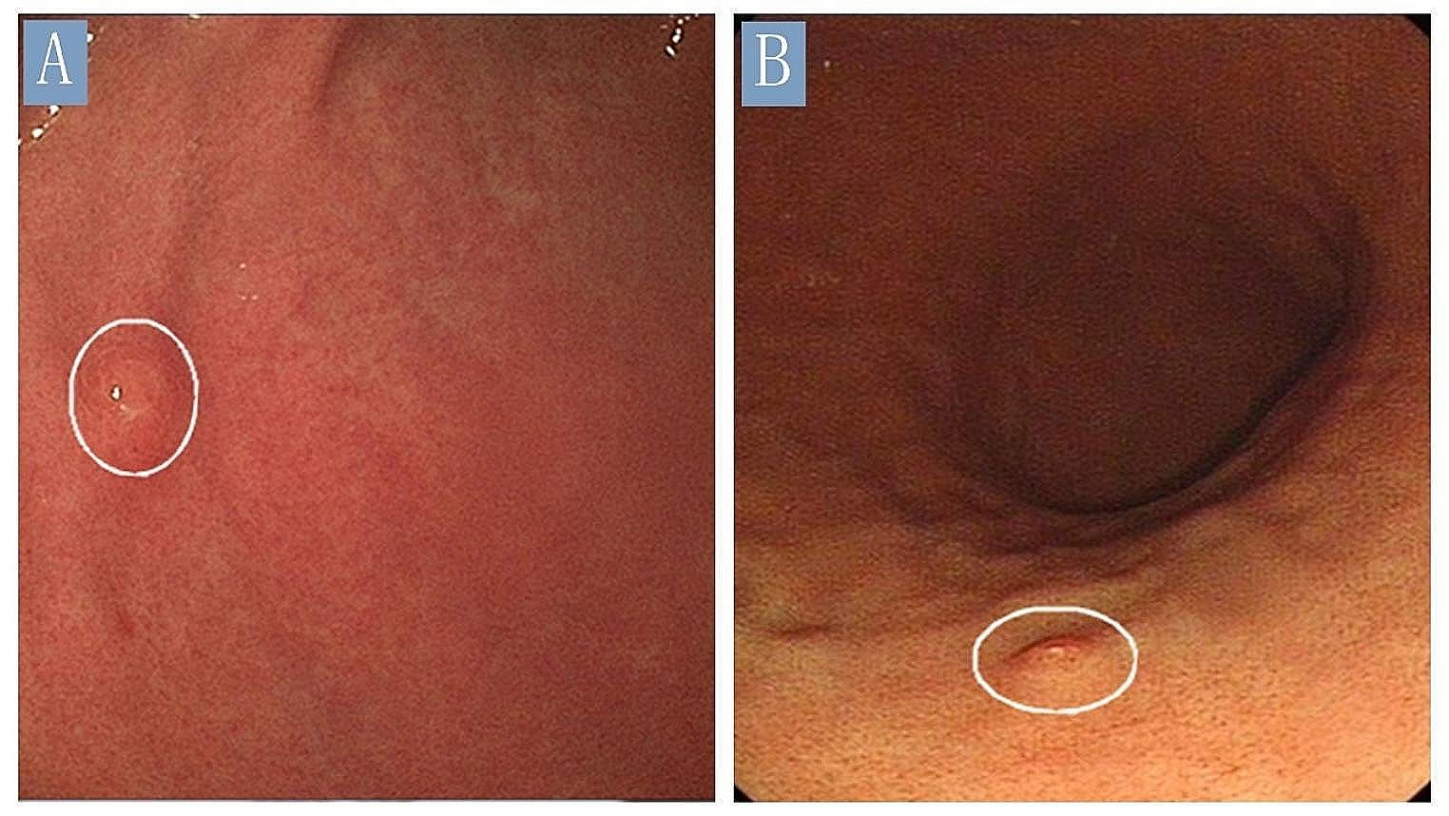
Endoscopic findings of gastric Langerhans cell histiocytosis. (**A**) Endoscopic examination revealed a flat polyp (arrows, size: 0.2 cm) that was located in the junction of the gastric body and antrum (Case 1). (**B**) Endoscopic examination revealed a reddish flat polyp with superficial erosion (arrows, size: 0.3 cm) that was located in the posterior wall of gastric body (Case 4)

Clinical data are summarized in Table 1. The affected patients ranged in age from 33 to 70 years (median, 46.5 years) and included one female and three males. Three patients presented with abdominal pain and the remaining patient was asymptomatic but had chronic atrophic gastritis and was identified during a follow-up examination. Endoscopic features were available for all patients. Three patients had solitary polyps or protruding lesions within the gastric body or at the junction of the gastric body and antrum (Fig. 1), whereas the remaining patient showed no abnormalities on gastroscopy. Cases 1 and 4 had multiple low-grade tubular colonic adenomas.

**Table 1:** Clinicopathological characteristics and molecular findings of adult isolated gastric LCH

Case	Age (years)/ Sex	Signs/ symptoms	Endoscopic finding/ Size(cm)	Location/ subtype	Treatment	Outcomes	Follow-up(months)	BRAF V600E mutation
Case 1 Present	33/M	Abdominal pain	Polyp (0.2)	JGBA/ unifocal	Biopsy	NED	4	Positive
Case 2 Present	43/M	Asymptomatic^#^	Normal	Gastric body/ unifocal	Biopsy	NED	36	Negative
Case 3 Present	52/M	Abdominal pain	Elevation (0.3)	Gastric body/ unifocal	Biopsy	NED	34	Negative
Case4 Present	70/F	Abdominal pain	Polyp (0.3)	Gastric body/ unifocal	Biopsy	NED	32	Negative
Case5^4^	47/F	Abdominal pain, nausea, diarrhea	Scattering erosions	Gastric body fundus/ SSM	Resection	NED	20	NA
Case6^5^	49/F	Asymptomatic	Sessile elevation	Throughout the stomach/ SSM	Biopsy	Spontaneous remission	66	NA
Case7^6^	48/M	Asymptomatic	Elevated reddish lesion (< 1.0)	Gastric body/ unifocal	Biopsy	No progression	12	NA
Case8^7^	51/M	Asymptomatic	Elevation (0.5)	Gastric antrum/ unifocal	Biopsy, ESD	NED	12	NA
Case9^8^	68/M	Dysphasia	Polyp	Gastric antrum/ unifocal	Biopsy	NED	22	NA
Case10^9^	29/F	Heartburn	Normal	Gastric body/ unifocal	Biopsy	NA	NA	Positive
Case11^10^	64/M	Asymptomatic	Elevated (1.0)	Gastric fundus/ unifocal	Biopsy, ESD	NED	6	NA
Case12^11^	37/M	Stomach discomfort	Erosion	Gastric body/ unifocal	biopsy	NED	14	Negative
Case13^12^	59/M	Abdominal pain	Elevation (1.0)	Gastric antrum/ unifocal	ESD	NED	4	Positive
Case14^13^	56/F	Asymptomatic	Elevation (0.5)	Gastric body/ unifocal	Biopsy	NED	3	Positive
Case15^14^	45/F	Asymptomatic	Polyp (0.6)	Gastric body/ unifocal	Biopsy, ESD	NED	3	Positive
Case16^15^	49/M	Abdominal distension and discomfort	Polyp (0.2)	Gastric body/ unifocal	Biopsy, ESD	NED	6	NA
Case17^16^	50/F	Helicobacter pylori-associated gastritis	Erythema	Gastric body antrum/ SSM	Biopsy	NA	NA	Positive
Case18^17^	23/M	Asymptomatic	Polyp (0.3)	JGBA/ unifocal	Biopsy	NED	6–60^*^	NA
Case19^17^	22/M	Hunger pain	Elevated erosive lesion	Gastric body/ unifocal	Biopsy ESD	NED		Positive
Case20^17^	46/M	Upper Abdominal distension	Polyp (0.3)	Gastric body/ unifocal	Biopsy	NED		Positive
Case21^18^	53/F	Abdominal discomfort	Multiple polyp (0.5–0.7)	Whole stomach/ SSM	Biopsy	Skin and bone involvement at 2 years	Alive at 30	NA

JGBA, The junction of the gastric body and antrum; NED, no evidence of disease; ESD, endoscopic submucosal dissection; NA, not available. SSM, single-system multifocal

^#^The patient had chronic atrophic gastritis without any clinical symptoms

*The article did not list the follow-up time for each case

### Pathological features

**Fig. 2:**
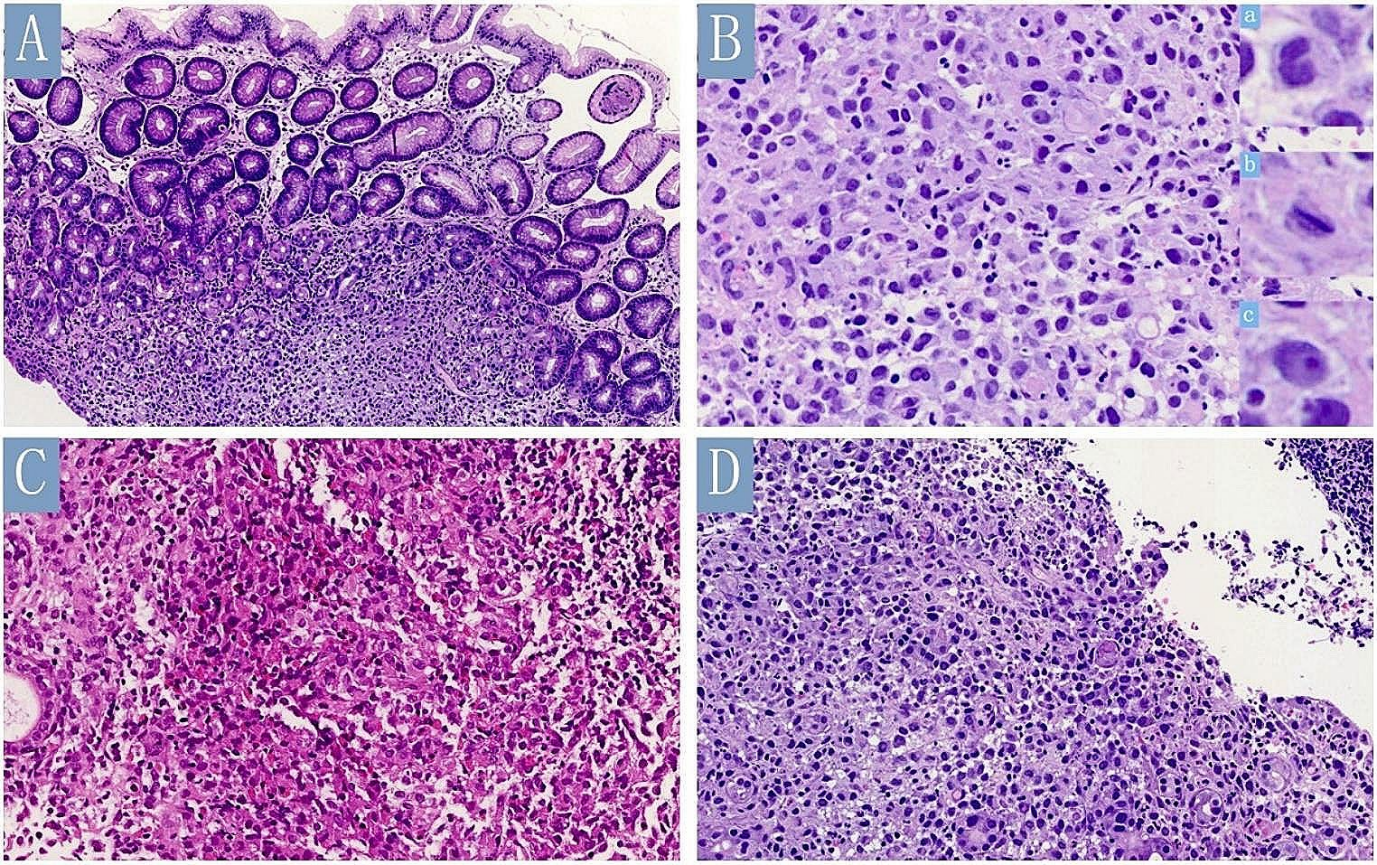
Histological findings of gastric Langerhans cell histiocytosis. (**A)** Histiocytoid cells infiltrating in a nested or sheeted pattern in the lamina propria of the gastric mucosa with an irregular boundary (Case 1) (hematoxylin and eosin, ×200). (**B**) The tumor cells were of intermediate size with indistinct cell borders, slightly eosinophilic cytoplasm, irregular or kidney-shaped nuclei (inset a), nuclear grooves (inset b), and occasional small nucleoli (inset c) (Case 1) (hematoxylin and eosin). (**C**) Eosinophils were the primary inflammatory cells in the background (Case 4) (hematoxylin and eosin, ×400). (**D**) Instead of an eosinophilic background, lymphocytes were the key inflammatory component (Case 1) (hematoxylin and eosin, ×400)

The lesions ranged in size from 0.2 to 0.3 cm and all were intramucosal. All cases showed destruction of the glands in the lamina propria of the gastric mucosa, with histiocytoid cells infiltrating in a nested or sheeted pattern (Fig. 2A). The tumor cells were of intermediate size with indistinct cell borders, a slightly eosinophilic cytoplasm, irregular or kidney-shaped nuclei, visible nuclear grooves, and occasional small nucleoli (Fig. 2B). In all cases, a variable number of mixed inflammatory infiltrates consisting of eosinophils, lymphocytes, plasma cells, and neutrophils were observed. Eosinophils comprised the majority of inflammatory infiltrates in three cases (Fig. 2C), whereas in the remaining case, lymphocytes were the predominant inflammatory components (Fig. 2D).

**Fig. 3:**
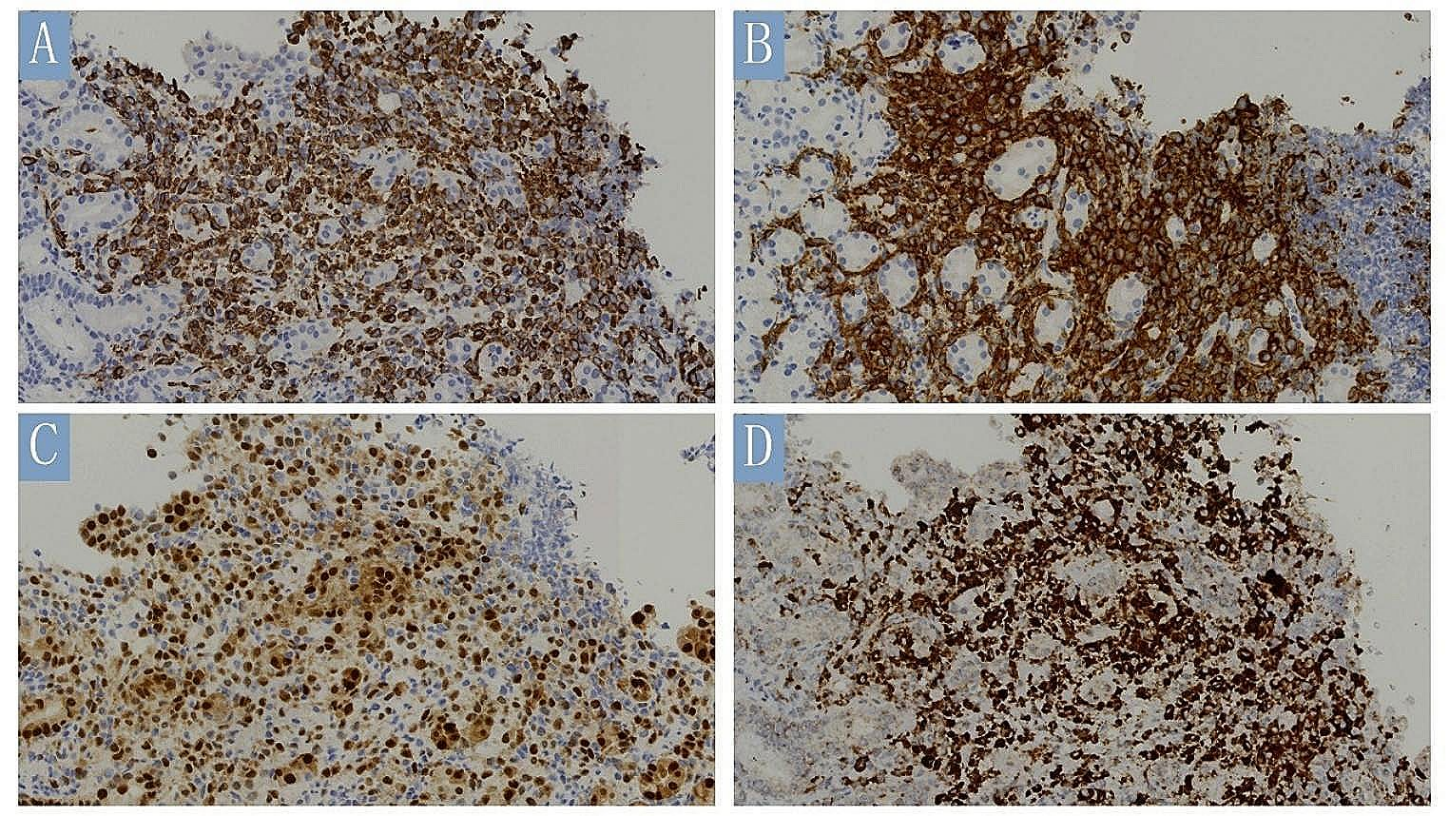
Immunohistochemical findings of gastric Langerhans cell histiocytosis in case 1. Tumor cells exhibited diffuse positivity to Langerin (**A**, IHC, ×400), CD1a (**B**, IHC, ×400), CyclinD1(**C**, IHC, ×400), and BRAF V600E (**D**, IHC, ×400)

Immunohistochemically, all four cases showed diffuse, strong staining for langerin (Fig. 3A), CD1a (Fig. 3B), and S-100, and patchy-to-diffuse positivity for CD68. CyclinD1 staining showed diffuse labeling in all cases (Fig. 3C). Staining for pan-cytokeratin and HMB-45 was negative in all cases.

Case 1 showed diffuse positivity for BRAF V600E (Fig. 3D). Cases 2, 3, and 4 were negative for BRAF V600E, and none of these cases showed mutations in *BRAF*, *KRAS*, or *NRAS* based on PCR test results. The Ki67 index ranged from 20 to 30%.

### Systemic inspection and follow-up

After the diagnosis of gastric LCH, all patients underwent a systemic examination to determine the extent of the lesion. Based on the clinical manifestations, physical examinations, and imaging examinations, none of the cases showed the involvement of other organs and systems, thus confirming the presence of isolated lesions. All patients were alive without recurrence or progression after 4–36 months of follow-up.

## Discussion

LCH, previously known as histiocytosis X, is a rare hematological disease with diverse manifestations in children and adults. In adults, LCH is grouped into four subtypes, unifocal (solitary lesion involving any organ), single-system pulmonary (isolated lung involvement, predominantly smoking-related), single-system multifocal (> 1 lesion involving any organ), and multisystem diseases (≥ 2 organs/systems involvement) [19], with the incidence of adult LCH being approximately 1–1.5/10^6^ per year [19–21]. Gastric LCH is mainly observed in pediatric patients with systemic diseases, whereas isolated stomach LCH in adults is extremely rare [22]. We identified 17 patients with solitary gastric LCH in the PubMed database after reviewing cases documented since 1983 [4–18], including four patients with single-system multifocal disease and 13 patients with unifocal disease based on the recognized subtypes of adult LCH [19] (Table 1). The age of the affected patients ranged from 22 to 68 years (median, 49 years), comprising seven females and 10 males. Among them, one patient had Helicobacter pylori-associated gastritis, seven patients were asymptomatic, and nine patients had gastrointestinal symptoms at presentation, including abdominal discomfort (three patients), abdominal pain (three patients), abdominal distension (two patients), nausea and diarrhea (one patient), heartburn (one patient) and dysphagia (one patient) (Table 1). Under endoscopy, 11 cases had solitary elevations or polyps (size 0.2–1.0 cm), two cases had multiple polyps or elevations, and other lesions included erosion (two cases) and erythema (one case), with only one case showing no lesions (Table 1). The most common site was the gastric body (eight cases), while other areas included the gastric antrum (three cases), gastric fundus (one case), gastric body and antrum (one case), gastric body and fundus (one case), junction of the gastric body and antrum (one case), and throughout the stomach (two cases) (Table 1).

This study included four patients diagnosed with stomach LCH with unifocal disease, based on the recognized subtypes of adult LCH [19]. They were aged 33–70 years, and included one female and three males. One patient had no corresponding gastrointestinal symptoms, and three patients presented with abdominal pain. Under gastroscopy, one patient showed no abnormalities, two patients had isolated polyps or protrusion of the gastric body, and one patient had isolated polyps at the junction of the gastric body and antrum. The clinical characteristics of the four patients were similar to those previously reported, but their age of onset was older, which expands the known age range of onset.

Gastric LCH is a rare condition. Pathologists may not have sufficient understanding of this condition, which can lead to missed diagnoses or misdiagnosis of other tumors. Additionally, the lack of specific clinical or endoscopic features further complicates accurate diagnosis. The correct diagnosis is based on micromorphological and immunohistochemical analyses of biopsies or postoperative specimens. Histologically, abnormally proliferative Langerhans cells are distributed in a nest- or sheet-like pattern, with medium-sized cells, irregular or renal nuclei, folded nuclear membranes, and longitudinal nuclear grooves. Most nucleoli are not obvious; however, the nucleolus is prominent in some cases. Further, the cytoplasm is rich and slightly eosinophilic. In the background, the infiltration of reactive inflammatory cells, such as eosinophils, lymphocytes, and plasma cells, may occur. In most cases, the inflammatory infiltration mainly involved eosinophils, whereas in rare cases, lymphocytes comprised the majority of infiltrating cells. Occasionally, focal necrosis and multinucleated giant cells have been seen to be present [4, 8, 19]. By immunohistochemistry, tumor cells specifically express CD1a, langerin, and S-100; thus, the minimal panel of antibodies should include those against langerin and CD1a [19]. As a downstream marker of the mitogen-activated-protein-kinase (MAPK) pathway, cyclinD1 is often activated in LCH. Several studies have confirmed that cyclinD1 nuclear staining is a useful diagnostic marker for excluding reactive Langerhans cell accumulation in the bone, lymph nodes, and skin lesions [23–25]. In our study, all four cases showed diffuse and strong cyclinD1 expression. Among the cases reported in the literature, five of seven tumors showed cyclinD1 expression.

The molecular events underlying LCH have been investigated. A series of studies confirmed that LCH can harbor a *BRAF V600E* mutation, other activating mutations in the MAPK-extracellular signal-regulated kinase (MAPK–ERK) pathway, and kinase fusions [26–28]. The *BRAF V600E* mutation was determined to be present in more than half of the LCH cases in the literature [26]. Among the 17 retrieved cases, only eight were subjected to *BRAF V600E* mutation testing, of which seven were found to have this mutation [9, 11–14, 16, 17]. In our cohort, four patients were tested for the *BRAF V600E* mutation, and three patients were simultaneously tested for mutations in *KRAS* and *NRAS* using a PCR detection method. We found that one patient had the *BRAF V600E* mutation based on positive IHC staining, while *BRAF*, *KRAS*, and *NRAS* mutations were absent in the remaining three cases. Based on previous cases and our four cases, the *BRAF V600E* mutation rate in adult gastric isolated LCH was determined to be 8/12. Of these, for the *BRAF V600E* mutation, three patients had weak expression and one patient had a negative result via IHC, but all were found to have a *BRAF V600E* mutation using molecular methods [12–14, 16]; thus, confirming a negative IHC test through one of the molecular methods is necessary. The *BRAF V600E* mutation can assist in the diagnosis of tumorigenic Langerhans cell proliferative lesions and may provide therapeutic targets for LCH patients with severe organ dysfunction [19].

Gastric LCH must be differentiated from several other conditions featuring histiocytic lesions, such as Rosai–Dorfman disease (RDD), Erdheim–Chester disease (ECD), and Langerhans cell sarcoma (LCS). RDD is characterized by large histiocytes with abundant light-stained-to-eosinophilic cytoplasm and characteristic emperipolesis. The nucleus is large and round, with vesicular chromatin, and a prominent nucleolus is present, with a lack of mitotic figures. Immunohistochemical features were positive for S100, fascin, and OCT2, but negative for langerin and CD1a [29]. ECD lesions exhibit bland xanthogranulomatous inflammation formed by foamy histiocytes, lymphocytes, plasma cells, eosinophils, and Touton giant cells, whereas histiocytes in ECD strongly express factor XIIIa and lack langerin and CD1a expression [30]. Moreover, LCS can be excluded based on the absence of overt malignant cytological features, such as significant cytological pleomorphism and increased mitotic activity. When making a differential diagnosis, a series of non-histiocytic tumors should also be considered, including systemic mastocytosis, malignant melanoma, and poorly differentiated carcinoma, all of which can be ruled out based on the characteristic morphological manifestations and negative immune expression patterns of CD1 and langerin.

The prognosis of LCH is related to the extent of the lesion, involvement of vital organs, and the response to treatment. The prognosis of single-system LCH is better than that of multisystem LCH, and patients with liver, spleen, and bone marrow damage have a higher risk of poor survival. The 5-year overall survival (OS) rate of patients with solitary LCH was > 90% [2, 3, 19]. Among the 17 previously reported cases of solitary gastric LCH, the treatment methods included surgical resection (one case), endoscopic submucosal dissection (six cases), and follow-up with conservative management (10 cases). Follow-up data were available for 15 patients. One patient with single-system multifocal disease had bone and skin involvement at 2 years of follow-up [18]. The other 14 cases had no gastrointestinal symptoms or disease progression during the follow-up period, which ranged from 3 to 66 months. In our group, all four patients were observed after diagnosis without other adjuvant treatments and had no symptoms for 4–36 months. Furthermore, in a series of 44 non-pulmonary unifocal LCH cases, 28% of patients (none with isolated gastrointestinal LCH) were reclassified as having single-system multifocal or multisystem disease at the last follow-up (median follow-up, 7.3 years), but were successfully treated, and the 5-year OS was 94% [31]. In another previous study, one patient with unifocal disease and one patient with single-system multifocal disease developed cutaneous disease 2 months and 24 months, respectively, after the initial diagnosis of lower gastrointestinal LCH, but the overall prognosis was favorable [8]. Therefore, local resection and observation are effective treatment methods for solitary gastric LCH, but careful long-term follow-up is needed to rule out multisystem diseases.

Of note, our study included a small number of cases and a short follow-up data period. In the future, studies with large sample sizes and long-term follow-up are still needed to evaluate the prognosis of solitary gastric LCH.

In summary, solitary gastric LCH is very rare, and most patients have no clinical symptoms or only mild gastrointestinal symptoms, with no specific manifestations on endoscopy. Pathologists should include LCH in the differential diagnosis to avoid a misdiagnosis or missed diagnosis. Additionally, a systemic examination should be conducted to determine the extent of the lesion, and long-term follow-up is necessary to monitor and rule out disease progression.

## References

[1] Harmon CM, Brown N (2015). Langerhans Cell histiocytosis: a clinicopathologic review and molecular pathogenetic update. Arch Pathol Lab Med.

[2] Goyal G, Acosta-Medina AA, Abeykoon JP (2023). Long-term outcomes among adults with Langerhans cell histiocytosis. Blood Adv.

[3] Sato A, Kobayashi M, Yusa N (2023). Clinical and prognostic features of Langerhans cell histiocytosis in adults. Cancer Sci.

[4] Nihei K, Terashima K, Aoyama K (1983). Benign histiocytosis X of stomach. Previously undescribed lesion. Acta Pathol Japonica.

[5] Iwafuchi M, Watanabe H, Shiratsuka M (1990). Primary benign histiocytosis X of the stomach. A report of a case showing spontaneous remission after 5 1/2 years. Am J Surg Pathol.

[6] Nozaki Y, Oshiro H, Nakajima A (2010). Image of the month. Langerhans cell histiocytosis of the stomach mimicking early gastric cancer. Clin Gastroenterol Hepatology: Official Clin Pract J Am Gastroenterological Association.

[7] Lee CK, Lee SH, Cho HD (2011) Localized Langerhans cell histiocytosis of the stomach treated by endoscopic submucosal dissection. Endoscopy 43 Suppl 2 UCTN: E268–E269. 10.1055/s-0030-1256605 10.1055/s-0030-1256605 21837608

[8] Singhi AD, Montgomery EA (2011). Gastrointestinal tract langerhans cell histiocytosis: a clinicopathologic study of 12 patients. Am J Surg Pathol.

[9] Sarbia M, Mauerer R, Bettstetter M (2015). Langerhans cell histiocytosis of the stomach with BRAF-V600E-mutation: case report and review of the literature. Z Gastroenterol.

[10] Lee SJ, Hwang CS, Huh GY (2015). Gastric Langerhans cell histiocytosis: Case Report and Review of the literature. J Pathol Translational Med.

[11] Yan F, Zhou Q, Gao Y (2018). Isolated langerhans cell histiocytosis of the stomach: a case report and literature review. Int J Clin Exp Pathol.

[12] Li YN, Shao SH, Zhao H (2020). Zhonghua Bing Li Xue Za Zhi = Chinese. J Pathol.

[13] Matsuoka Y, Iemura Y, Fujimoto M (2021). Upper Gastrointestinal Langerhans Cell histiocytosis: a report of 2 adult cases and a literature review. Int J Surg Pathol.

[14] Wang L, Yang F, Ding Y (2022). Gastrointestinal langerhans cell histiocytosis with unifocal, single-system involvement in adults: cases report and literature review. J Clin Lab Anal.

[15] Zhou XL, Fan L, Gu WX (2022). Zhonghua Bing Li Xue Za Zhi = Chinese. J Pathol.

[16] Mora LB, Hough M, Moscinski L (2023). Incidental gastric langerhans Cell histiocytosis and synchronous adenocarcinoma of the Colon: an interesting Case Report and Literature Review. Cancer Diagnosis Prognosis.

[17] Chen X, Yuan JP, Zhao LN (2023). Zhonghua Bing Li Xue Za Zhi = Chinese. J Pathol.

[18] Wada R, Yagihashi S, Konta R (1992). Gastric polyposis caused by multifocal histiocytosis X. Gut.

[19] Goyal G, Tazi A, Go RS (2022). International expert consensus recommendations for the diagnosis and treatment of Langerhans cell histiocytosis in adults. Blood.

[20] Goyal G, Shah MV, Hook CC (2018). Adult disseminated Langerhans cell histiocytosis: incidence, racial disparities and long-term outcomes. Br J Haematol.

[21] Makras P, Stathi D, Yavropoulou M (2020). The annual incidence of Langerhans cell histiocytosis among adults living in Greece. Pediatr Blood Cancer.

[22] Yadav SP, Kharya G, Mohan N (2010). Langerhans cell histiocytosis with digestive tract involvement. Pediatr Blood Cancer.

[23] Ben Rejeb S, Charfi L, Sahraoui G (2021). Cyclin D1: potential utility as marker for Langerhans cell histiocytosis. J Immunoassay Immunochem.

[24] Chatterjee D, Vishwajeet V, Saikia UN (2019). CyclinD1 is useful to Differentiate Langerhans cell histiocytosis from reactive langerhans cells. Am J Dermatopathol.

[25] Shanmugam V, Craig JW, Hornick JL (2017). Cyclin D1 is expressed in neoplastic cells of Langerhans Cell histiocytosis but not reactive Langerhans Cell Proliferations. Am J Surg Pathol.

[26] Badalian-Very G, Vergilio JA, Degar BA (2010). Recurrent BRAF mutations in Langerhans cell histiocytosis. Blood.

[27] Chakraborty R, Hampton OA, Shen X (2014). Mutually exclusive recurrent somatic mutations in MAP2K1 and BRAF support a central role for ERK activation in LCH pathogenesis. Blood.

[28] Durham BH, Lopez Rodrigo E, Picarsic J (2019). Activating mutations in CSF1R and additional receptor tyrosine kinases in histiocytic neoplasms. Nat Med.

[29] Ravindran A, Goyal G, Go RS (2021). Rosai-Dorfman Disease displays a unique monocyte-macrophage phenotype characterized by expression of OCT2. Am J Surg Pathol.

[30] Goyal G, Heaney ML, Collin M (2020). Erdheim-Chester disease: consensus recommendations for evaluation, diagnosis, and treatment in the molecular era. Blood.

[31] Hu M, Goyal G, Abeykoon JP (2022). Clinical features and outcomes of non-pulmonary unifocal adult Langerhans cell histiocytosis. Blood cancer J.

